# Sequential patterns of spikes and scale-invariance in modular networks

**DOI:** 10.1186/1471-2202-15-S1-P225

**Published:** 2014-07-21

**Authors:** Timothee Leleu, Kazuyuki Aihara

**Affiliations:** 1 Institute of Industrial Science, The University of Tokyo, 4-6-1 Komaba, Meguro-ku, Tokyo 153-8505, Japan

## 

**Figure 1:**
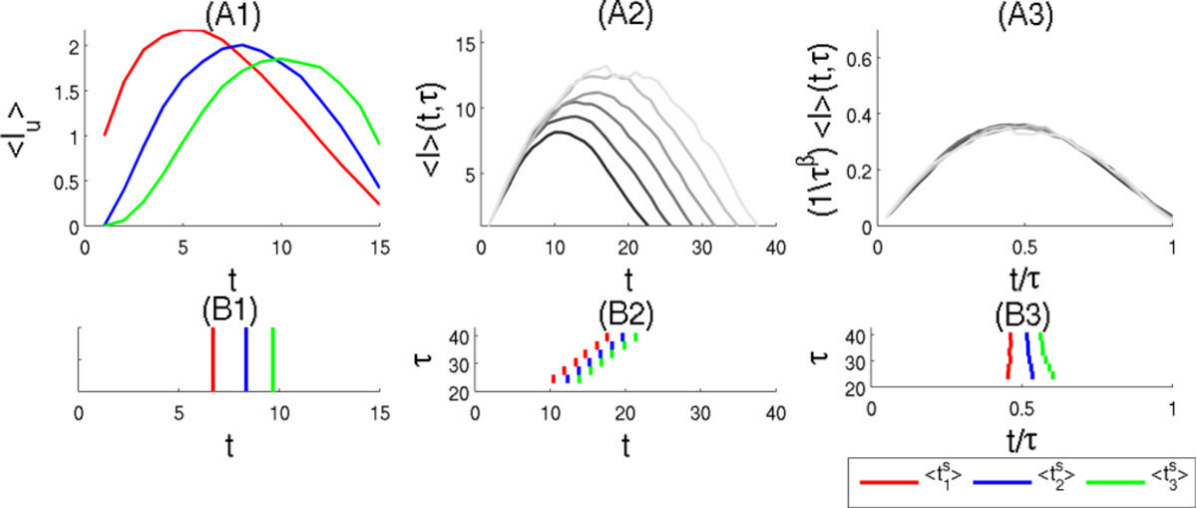
**(A1) The average number of neurons $<l_{u}\left(t\right)>$ spiking at the time-step  t in the sub-networks u=1, 2, and 3 are shown red, blue, and green, respectively, for avalanches of duration τ=15 starting from the sub-network 1**. (B1) The same as (A1) for the mean spiking time $<{t_{u}}^{s}>$. (A2) Cumulated temporal profiles $<l\left(t,\tau \right)>=\underset{u\in S}{\sum }<l_{u}\left(t\right)>$ for avalanches of durations τ∈T={20,23,26,29,32,35,38}. (B2) The same as (B1) for avalanches of durations τ∈T. (A3) and (B3) are the same as (A2) and (B2), respectively, after rescaling the axes (β=1).

It has been reported that there are consistent sequential patterns of spikes after the transitions to the up state during slow wave sleep[1]. The up states may be characterized by critical dynamics[2] for which the avalanche sizes distribution is scale-invariant[3]. In order to understand the mechanism of the sequential patterns, it may thus be necessary to study the fine structure of avalanche transmission between multiple neuronal ensembles at criticality. We have developed an analytical model of avalanche dynamics in modular networks. The univariate distribution of avalanche sizes[4] can be extended to a joint probability distribution $P^{w}\left({\left\{L_{u}\right\}}_{u\in S}\right)$ describing the probability that $L_{u}$ neurons are active in each sub-network  u during avalanches that start from the sub-network  w with  u∈S={1,...,M} and  M the number of sub-networks. The mean temporal profile[3]$<l_{u}\left(t\right)>$ of the avalanches in each sub-network  u can then be detailed (see Figure 1 (A1) and (B1)) and are reminiscent of the temporal patterns observed experimentally. The sequential patterns depend on the average connection strengths between the sub-networks. At criticality, the cumulated temporal profiles can be collapsed using standard rescaling of the axes and yield a single universal scaling function[3] (see Figure 1 (A2) and (A3)). After rescaling, the mean spiking times $<t_{u}^{s}>$ in each sub-network are functions of the durations of the avalanches. In Figure 1 (B3), the intervals between successive mean spiking times $<t_{u+1}^{s}>-<t_{u}^{s}>$ are proportionally shorter for longer avalanches near criticality.
